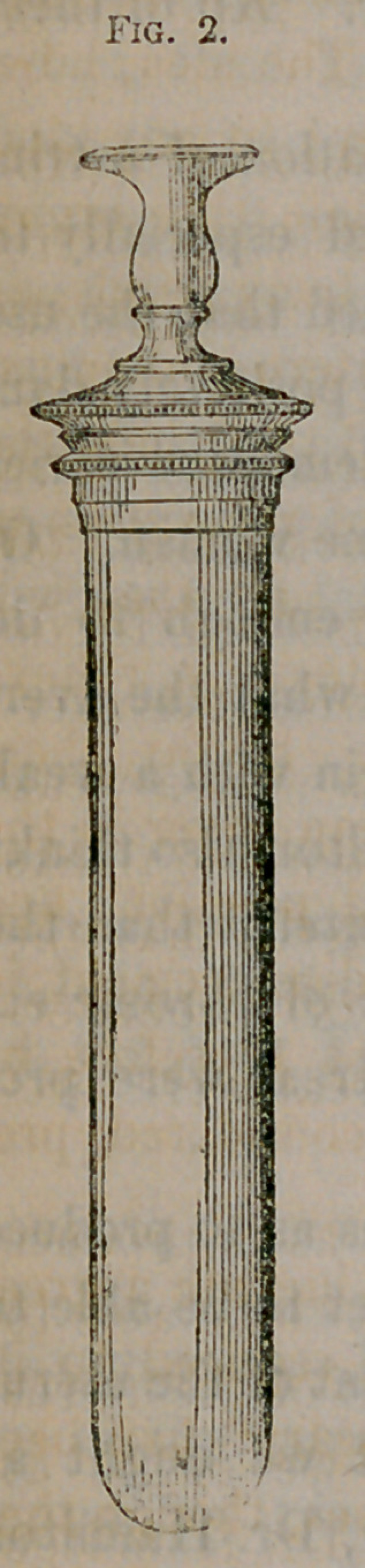# Lectures on Displacements of the Uterus

**Published:** 1860-04

**Authors:** E. R. Peaslee

**Affiliations:** Professor of Obstetrics and Diseases of Women and Children in the New York Medical College


*Lectures on Displacements of the Uterus.* By E. R. PEASLEE, M.D., LL.D., Professor of Obstetrics and Diseases of Women and Children in the New York Medical College.

(Continued from last No. of the MONTHLY.)

LECTURE No. II.

GENTLEMEN—In the preceding lecture I have spoken of the structure of the uterus, its normal position and relations, and the agencies maintaining it in position; and in general, also, of the causes of uterine displacements, their symptoms, the methods of recognizing them, and their prognosis. I shall to-day speak in a general way of certain means adopted in the treatment of this class of diseases; and also call your attention to the nervous endowments of the uterus, as explanatory of the manifold symptoms already enumerated.

I. The general indications in the treatment of uterine displacements are:

1st. To replace the uterus.

2nd. To maintain it in its normal position by such aids as may be either temporarily or permanently required.

The former of these objects is accomplished by manipulations, either alone or combined with the use of instruments; the latter, by various means, among which the use of astringents *per vaginam*, and of mechanical supports, are most prominent. And these two classes of remedial means will next be considered.

A. Of *astringents*, the tannic acid, alum, sulphate of zinc or copper, nitrate of silver; or infusions of vegetable substances containing the first, as oak-bark, nutgalls, matico, &c., are most commonly used. All these are used most frequently as vaginal injections, and therefore in solution in water; though they may also be used to advantage in the form of suppositories, or ointment, or of powder. All of them are also used in several other forms of uterine disease.

Some writers, however, object *in toto* to the application of astringents *per vaginam;* though such are generally opposed especially to astringent vaginal injections. Dr. Hamilton maintained that the use of strong astringent injections is injurious, and may be positively dangerous; since the irritability of the vaginal mucous membrane varies in different women, and at different times in the same woman. Of course it is admitted that an injection may be strong enough to do more harm than good in any case. Experience shows what the average strength should be; and also, that we should begin with a weak and proceed to a stronger application. But Dr. Hamilton also thinks that astringent injections are more apt to injure the uterus than the vagina itself; and asserts that most of the many cases of chronic enlargement of the uterus he had been called upon to treat were produced by their use.

Of course, astringents may be so used in some cases as to produce this result. But we have only to be aware of this fact to be able to guard against it. They can produce chronic enlargement of the uterus only by first producing inflammation; and this effect we ought at once to detect, and then to remove the cause. Finally, Dr. Hamilton asserts that a sudden suppression of an habitual vaginal discharge by the use of astringents is often "followed by distressing headache, obstinate inflammation of the eyes, or eruptions on the face."*

* Practical Observations, p. 17.

But, admitting that all the preceding consequences may result from the indiscriminate use of astringent vaginal injections, they will often be found to be very beneficial, if used in accordance with the following directions:

1. Withhold them entirely if there is any inflammation, or even much congestion of the vagina or the uterus.

2. Use them of the proper strength, beginning with a weak application.

3. In case of a profuse discharge, never check it suddenly, by their use.

4. Discontinue the application, if any bad effect ensues from it.

Used with these precautions, I regard astringent vaginal applications, in the various ways enumerated, as very valuable aids in the treatment of the displacements under consideration, as well as of several other uterine diseases; and I shall have occasion, therefore, to recommend them further on.

Fig. 2.

I have insisted on beginning with a weak application. I have been in the habit, on first prescribing an astringent injection, to direct the patient to mix a portion of the whole amount prescribed (say one-fourth) with as much water, and use that first; then mix another portion with half as much water; and next apply the full strength. An ounce of the astringent solution is sufficient for each application, if properly applied. There is no need of one half pint to a pint, as is sometimes directed. It should be used two or three times daily, and applied *cold*. These injections are, however, too often ineffectual, from a wrong method of administration. It is not enough to make your prescription, and tell the patient to use it as an injection. She must be told what sort of an instrument to obtain, and how to use it.

The best vaginal syringe is the "India-rubber Vaginal Syringe, manufactured by the Beacon Dam Company." It is made of vulcanized gum-elastic, is not acted on by acids, is strong, light, and elegant, and of the best form. There are three sizes of this instrument, and the accompanying cut gives an idea of its form—being one-third size linear. A glass instrument of the same form is of course less expensive, and answers very well as a substitute. The instrument being selected, the patient is to be instructed to use first an injection of cold water, (half pint or more,*) and then apply the astringent injection while in a recumbent position, with the pelvis slightly elevated; using but a single syringeful, but retaining the instrument two or three minutes, at the same time preventing the escape of the fluid from the vagina, so far as may be. If water is not previously used as just advised, the secretion, coagulated by the astringent, accumulates in the vagina, and produces irritation; or at least protects the vaginal membrane from the contact of the astringent, and thus entirely defeats the object of its administration.

Astringent suppositories may also be applied by the patient herself; but in the form of powders the application must be made by the practitioner, through the speculum.

B. *Mechanical supports* may be arranged in two classes—the *external* and the *internal.*

The *external* include a great variety of bandages, most of which are termed abdominal and utero-abdominal supporters. The idea, however, of supporting the uterus (except during pregnancy) by any kind of bandage applied around the abdomen or pelvis, while this organ does not rise to the level of the superior plane of the bony pelvis, as I have shown, (Fig. 1.) is simply absurd. The term "utero-abdominal supporter" is therefore a misnomer, and only calculated to delude the ignorant. Abdominal supporters are useful in cases of relaxation of the abdominal walls, during pregnancy, or otherwise, and they will sometimes, by their pressure, either anteriorly or posteriorly, relieve some symptoms produced by a uterine displacement. So far, then, they are of real value. But on the other hand, they can never in the least tend to restore a displaced uterus to its normal position; and they may, by their indirect pressure upon it, insure a displacement in cases with a mere predisposition thereto. They must therefore be used with extreme caution in the displacements to be considered, provided other complications render their use expedient.

The *internal,* or intra-vaginal mechanical supports, include a great variety of appliances to support the uterus after it is reduced to its normal position, and to which the general term "pessary" has been applied. Some, however, object to pessaries in all cases, though they use a ball of lint, soaked in an astringent solution, instead. Such a ball, however, when used to support the uterus, is simply a medicated pessary. So, also, is a muslin bag, filled with an astringent powder,

* The same vaginal syringe may be used for this purpose.

when used for a similar purpose. The ancients used a variety of vegetable substances, and other matters too disgusting to be named, as pessaries. I shall have occasion to recommend the two forms of medicated pessary just mentioned in another connection.

But the word "pessary" is usually applied to an instrument made of wood, ivory, glass, gum-elastic, gutta-percha, cork, sponge, or some metal that is not liable to oxydation; of a circular or oval outline; sometimes discoid, and perforated in the centre; sometimes in the form of a simple ring, or of a globe, or an ovoid—and which is introduced into the vagina to support the uterus, as before explained. Within a few years some practitioners have also used instruments passing partly into the cavity of the uterus, in the treatment of uterine displacements, and these may be termed *intra-uterine* pessaries. These are, however, recommended only in cases of anteflexion or of retroflexion, and I shall speak of them in connection with these two displacements. We should, however, form a definite idea of the actual value of intra-vaginal pessaries in this connection.

I would as soon say I would never use a splint in surgical practice, as that I would never use a pessary in the treatment of uterine displacements; though they are not often, like splints, quite indispensable to the treatment of the case. They are, however, in many cases the best remedy, if used with the proper restrictions, and therefore should be preferred to all other means.

Among the high authorities opposed to the use of pessaries are Dr. Hamilton, before quoted, Dr. Rigby, and the late Prof. Dieffenbach; and the following is the sum of their objections to them :

1. They are indelicate for the patient to wear.
2. They cause a (sometimes even putrid) vaginal discharge, by irritating the vaginal mucous membrane.
3. They are of no use, or even fall out, if too small; and dilate the vagina if too large.
4. They cause ulceration and fungous growths, and ultimate contraction of the vagina.
5. They produce constipation and irritation of the bladder.
6. They subject the patient for life to the charge of the medical attendant.
7. They may become incrusted by calcareous matter, and have to be broken before they can be removed; or may pass, by ulceration, through from the vagina into the bladder or rectum.

If but a small part of these effects of the use of pessaries be unavoidable, very few practitioners certainly would have the hardihood to use them, even in desperate cases. But this is by no means the fact; and to denounce an instrument because it may be made to do mischief is hardly philosophical. The question should be, Can the instrument be made to do good? and if so, under what conditions, and with what restrictions? And, as furnishing a reply to the latter question, I should say that the following rules should, in practice, be adopted:

1. Pessaries are not to be used if there be irritation or inflammation of the vagina or the cervix uteri, or any other organic disease of the latter. If neither of these exist, the question of their use may be considered.

2. Their size must be precisely adapted to the cases requiring them.

3. Watch the patient after the introduction of the instrument, and remove it if any occasion for so doing arise.

4. Examinations should subsequently be made at proper intervals to ascertain if any mischievous effects are being produced, in which case the instrument is to be removed.

5. The woman must be taught to keep the instrument cleansed by daily vaginal injections of water or weak soap-suds. I once removed a glass pessary which had remained fourteen months in the vagina. It had not caused the least irritation, though the physician who introduced it had not instructed the patient to take the precaution I have just inculcated.

6. Remove the instrument occasionally, (once a month, at least,) to make sure that it remains in good condition; reapplying it if it does so remain, or a smaller one, if such will now answer.

I consider the question of the delicacy of wearing a pessary as too trivial for serious consideration. If notions of delicacy alone were to control us, we should never even ascertain the existence of these displacements by any examination, nor advise any local treatment at all if they were conjectured or discovered.

Used with the precautions above mentioned, and in cases requiring mechanical support, I consider pessaries temporarily used to be of great value; and I shall here add some remarks upon their composition and their forms.

In order to answer the conditions before specified, a pessary should be (1) as light as possible; (2) made of a material which will retain a smooth surface, and therefore not be acted upon by the uterine or vaginal fluids; and (3) be of a form best adapted to the vaginal walls, and causing the least possible pressure consistent with securing the object for which it is used.

The *form* which insures the greatest degree of lightness is the ring, and I therefore prefer it. If the material be glass, it is liable, unless quite thick and heavy, to fracture. But the ring pessary may be made of a thin plate of silver or gold, first soldered in the form of a tube, and then curved so as to form a circle of the proper diameter. A solid ring of ivory may answer, but is heavy, and ivory is objectionable as a material. Gum-elastic renders the vaginal discharges intolerably fœtid. Gutta percha is not, however, liable to this objection, (if used in its usual purity,) and I prefer, for most purposes, the pessary I have so often used before you, consisting of a ring, $2\frac{1}{4}$ inches (more or less) in diameter, made of a piece of watch-spring, which is completely covered by gutta percha, so as to make the ring about four lines thick. This ring pessary has the advantage of being light, and can be made to maintain a circular or elliptical form at pleasure. Besides, it is not acted upon by the fluids in contact with it, if ordinary care is exercised to keep it clean. A patient of mine retained one eleven months —though contrary to my directions—and at the end of that time its surface had not changed in the least. Their size will vary from $1\frac{1}{2}$ to 3 inches in diameter, in different cases. A simple ring of tin is also a very serviceable, and a cheaper pessary; and being quite flexible, and rather firm, it may be bent into any required form before its introduction, and which it will afterwards retain. A ring pessary, properly adjusted, will answer all the requirements of this class of instruments, in almost every case, except when a globe pessary is needed.

The pessary just described is used to retain the uterus after it is replaced; the *globe* pessary is an important aid, in some cases, in replacing and then retaining the uterus. It finds its peculiar value in some cases of prolapsus uteri, with adhesions of this organ, as will be seen. It is simply a hollow sphere, or is sometimes shaped like an egg, and made of a very thin and light plate of gold, or of silver. A solid ball of any substance is objectionable, on account of its weight.

Of *stem pessaries* I have little to say. They are kept in the vagina by some external apparatus; which should always be avoided, if possible, as very inconvenient to the patient; and I have not, of late, met with any case in which I could not accomplish all that this form of instrument promises, by the use of the forms already described. For it is only in prolapsus that the stem pessary pretends to any superior merit. I should, however, add, that the stem pessary may be the best in certain complications with prolapsus, hereafter to be specified. Sponge pessaries become intolerably offensive, even in a few hours, and cause much irritation of the vaginal membrane. Cork is also liable to the same objection in a less degree. Of late, therefore, I have confined myself, with very rare exceptions, to the ring pessary, made of gutta percha or metal, and the globe pessary of metal, as before described; with such medicated pessaries as will be specified further on. I long ago laid aside the gum-elastic bags, introduced into the vagina in a collapsed state, and subsequently inflated; they becoming intolerable from the odor they impart to the vaginal secretion.

II. In regard to the *nervous endowments* of the uterus, quite diverse opinions have been recently maintained. Dr. Robert Lee published the results of his investigations in 1842, (see Philosophical Transactions for that year,) and maintained that an extensive nervous network covers the entire uterus; and which is derived from the hypogastric and spermatic plexuses, and forms vesical and vaginal ganglia, and anterior and posterior sub-peritoneal ganglia and plexuses. He also maintained that this great system of nerves enlarges during gestation, and returns again to its former condition after parturition. Most obstetricians adopted Dr. Lee's ideas on this subject till within the last few years. But Dr. Lee did not verify his dissections by the use of the microscope; and Dr. Snow Beck, applying that test, has come to the conclusion that the uterus is, on the contrary, not abundantly supplied with nerves. I have no difficulty in believing that Dr. Beck is in this correct; for, in addition to his positive demonstrations, the uterus gives no evidence of a very high degree of innervation either in its physiological condition or its pathological states. That the nerves are, however, enlarged during gestation, is certainly in the highest degree probable. All our certain knowledge, however, up to the present time, respecting the sources of the nerves distributed to the uterus and its appendages, may be expressed in a rapid and general manner as follows:

The genital organs of the human female derive their spinal nerves from the lumbar and the sacral plexuses; and their sympathetic branches from the spermatic and the inferior hypogastric (or pelvic) plexuses.

A. The *spinal nerves* from the lumbar plexus are as follows: The scrotal branch of the *ilio-scrotal* nerve (as it is called in the male) is distributed to the round ligaments and the labia majora; while its abdominal branch goes in part to the mons veneris and to the groin. A branch of the *ilio-inguinal* nerve also communicates with the latter, and proceeds to the same parts. The genital branch of the *genito-crural* nerve is also distributed to the round ligaments and the labia majora, and to the skin of the groin. Other branches from the lumbar plexus are distributed to the integuments and muscles of the thigh, and the internal saphena branch of the femoral (or anterior crural) nerve, even to the foot. These facts account for the reflex pain often felt by patients with affections of the uterus, in parts at a distance, and especially in the lower extremities.

The following are the *spinal* nerves from the *sacral plexus:* The *visceral nerves* ascend on the sides of the vagina, rectum, and bladder, and the 3d and 4th sacral nerves give off branches to the uterus. The superior branch of the *internal pudic* nerve goes mainly to the clitoris; its perineal branch to the vulva and the perineum. The perineal cutaneous branch of the lesser ischiatic nerve also supplies the vulva.

B. The *sympathetic* nerve-fibres are derived from the following sources: The *spermatic plexus* gives off branches to the round ligaments, the ovaries, the Fallopian tubes, and the uterus itself. The *inferior hypogastric* (or pelvic) *plexus* gives off branches to all the organs in the pelvis. The *uterine nerves* come off above the sacral nerves, and thus penetrate the substance of the whole of the uterus in company with its arteries. They therefore consist mostly of sympathetic fibres. For you are aware that the sympathetic nerves, so called, contain also some coarse (or spinal-nerve) fibres. On the other hand, the nerves to the vagina from the vaginal plexus (an offset from the pelvic) contain many coarse, (or spinal,) and but few fine (or sympathetic) fibres. This accounts for the greater sensibility of the latter portion of the genital apparatus.

A recapitulation of the preceding facts gives the following result:

1. The *uterus* is supplied with *spinal* nerve-fibres from the sacral plexus, and with *sympathetic* fibres from the spermatic and the pelvic plexuses. As the visceral nerves are also distributed to the other organs of the pelvis, and other branches from the sacral plexus are sent to the gluteal and sacral region, and to the lower extremity, we should expect a wide range of reflex sensations developed in the various uterine affections. Moreover, this expectation is further enhanced by the fact that the pelvic plexus also gives off branches to the other organs in the pelvis, as well as to the uterus. Reaching the uterus between the two layers of the broad ligament, its nerves are distributed from the fundus and body to the neck, and to the latter part most abundantly. More branches are also sent to its posterior than to its anterior half, and hence the former is its most sensitive portion. Besides, the body of this organ is not so highly sensitive in its normal condition as the neck, from the greater proportion in its nerves of the fine fibres.

2. The *appendages* of the uterus are supplied with nerves as follows: (1.) The *ovaries* receive branches from the spermatic plexus alone, so far as is known; though there are doubtless some spinal nerve-fibres in the branches sent to them. The uterus also receives branches from the same plexus, as we have seen; and hence a direct sympathy between the uterus and the ovaries, both in their physiological and in their pathological states. (2.) The *Fallopian tubes* receive branches from the spermatic plexus also, and a branch from one of the uterine nerves. Thus, they sympathize with the ovaries on the one hand, and the uterus on the other. Like the ovaries, they are not known to receive any distinct branches of spinal nerves. The importance of this triple sympathy in relation to the fecundation of the ovum, and its subsequent transmission through the Fallopian tube into the uterus, will not be overlooked. (3.) The *round ligaments* are supplied from the lumbar plexus and the spermatic plexus.

3. The *vagina* is supplied with nerves from the sacral and the pelvic plexus. It has already been stated that even in the sympathetic branches from the latter plexus, the spinal nerve-fibres predominate, and hence its higher degree of sensibility as compared with the uterus. The *vulva* is supplied from both the lumbar and the sacral plexus.

But this must suffice on this topic. My object is to prepare you to expect a variety of pains, and other modifications of sensibility, in cases of disease of the uterus and its appendages; for since, as we have seen, a direct sympathy of the uterus itself with all its appendages exists on the one hand—while, on the other, there is a sympathy between each of these and more distant parts deriving their nervous endowments from the same source—we can never predict precisely what or how many peculiar sensations may be developed in any particular case; and we may perhaps have the same symptoms in cases quite unlike.

And you will now, I trust, more fully appreciate the statement, since you can give a reason for it, that many symptoms may be common to a great variety of uterine affections; that the mere rational signs of uterine displacements are not at all reliable; and that a vaginal examination alone can lead to a *positive* diagnosis.

Prolapse of the uterus will be the subject of my next lecture.

## Figures and Tables

**Fig. 2. f1:**